# Blind Recognition of Frame Synchronization Based on Deep Learning

**DOI:** 10.3390/s24206767

**Published:** 2024-10-21

**Authors:** Jiazheng Wei, Shitian Zhang, Mingchao Jin, Xiandeng He, Dongxiao Quan, Chen Chen

**Affiliations:** 1 School of Telecommunication Engineering, Xidian University, Xi’an 710126, China; 22011210729@stu.xidian.edu.cn (J.W.); 21011210111@stu.xidian.edu.cn (M.J.); xdhe@mail.xidian.edu.cn (X.H.); cc2000@mail.xidian.edu.cn (C.C.); 2 National Key Laboratory of Electromagnetic Environment, China Research Institute of Radiowave Propagation, Qingdao 266108, China; shitian_zhang@126.com

**Keywords:** frame synchronization, blind recognition, deep learning, ResNet, non-cooperative communication

## Abstract

In this paper, a deep-learning-based frame synchronization blind recognition algorithm is proposed to improve the detection performance in non-cooperative communication systems. Current methods face challenges in accurately detecting frames under high bit error rates (BER). Our approach begins with flat-top interpolation of binary data and converting it into a series of grayscale images, enabling the application of image processing techniques. By incorporating a scaling factor, we generate RGB images. Based on the matching radius, frame length, and frame synchronization code, RGB images with distinct stripe features are classified as positive samples for each category, while the remaining images are classified as negative samples. Finally, the neural network is trained on these sets to classify test data effectively. Simulation results demonstrate that the proposed algorithm achieves a 100% probability in frame recognition when BER is below 0.2. Even with a BER of 0.25, the recognition probability remains above 90%, which exhibits a performance improvement of over 60% compared with traditional algorithms. This work addresses the shortcomings of existing methods under high error conditions, and the idea of converting sequences into RGB images also provides a reliable solution for frame synchronization in challenging communication environments.

## 1. Introduction

In modern digital communication systems, frame synchronization is typically achieved by periodically inserting predefined synchronization code into the binary data stream. The recognition of synchronization codes is a prerequisite for subsequent signal processing tasks such as error correction and information extraction. The receiver of the cooperating party can utilize a suitable algorithm to locate the known synchronization codes easily [1,2]. In contrast, the non-cooperative receiver needs to first obtain synchronization codes form the intercepted signals with little prior information, and then they can further get the original binary data stream, which is a more complicated process compared to the collaborative scenario [3,4,5]. The frame synchronization recognition includes obtaining the length of the predefined synchronization code, the specific synchronization code as well as the frame length, which is defined as the intervel between the adjacent synchronization codes.

In the classical frame synchronization blind recognition algorithms [6,7,8,9,10,11,12,13], Wang et al. first utilized small-area detection to estimate the frame length [6]. According to the characteristics of QPSK signal, Qin et al. proposed a recognition algorithm based on soft-decision [7], which achieved better performance of synchronization code recognition than hard-decision. In 2017, He realized the estimation of frame parameters by constructing a hierarchical model of equal frame length and rank finding in small regions [8]. In 2019, Xu et al. proposed an improved frame synchronization recognition algorithm based on self-correlation, which achieves reasonable frame length estimation by introducing peak-to-average ratio (PAR) [9]. In 2020, Shao and Lei used triple correlation filtering to identify the frame length after eliminating the redundant data of the filling matrix, calculated correlation values to extract the key fields, and finally extracted the synchronization code through dispersion analysis [10]. Chen et al. constructed matrices with varying column numbers and computed the first-order cumulant of these matrices. They set decision thresholds based on the frame structure characteristics to distinguish between synchronous bit columns and data bit columns, thereby achieving the recognition of frame length and synchronization codes [12]. In 2023, Jin utilized high-order cumulant to detect the burst signal in time domain, and then completed the frame synchronization recognition by combining the code domain information [13]. Some researchers have utilized the encoding features of signals in their recognition of frame synchronization [14,15,16]. Xia and Wu have achieved the blind identification of encoders and blind estimation of delays by maximizing the average log-likelihood ratio [15]. In 2023, Feng et al. have accomplished blind frame synchronization by utilizing soft information from frozen bits and employing polar coding assistance [16]. Wang et al. improved upon the pattern matching algorithm by removing the bad character array table from the preprocessing phase and retaining only the good suffix character array table. Additionally, to address the issue of the pattern matching algorithm being limited to exact matching, the proposed algorithm introduced error tolerance, enabling fuzzy matching for bit stream data. It also modified the index shifting rules for matching, which helped avoid frame header loss due to bit errors caused by noise in the communication environment, resulting in faster frame header detection speeds and improved bit error rate performance [17]. In 2024, Wang et al. divided the bitstream evenly into multiple windows, and perform a sliding XOR operation on the first two windows to obtain the Extended Synchronization Word (E-SW). Then, use the obtained E-SW to perform a sliding correlation operation with the remaining windows, resulting in the corresponding E-SWs for each window, which form the E-SW set. Finally, conduct statistical analysis on the E-SW set to filter out the codewords of the unknown synchronization word [18]. Lv et al. studied a joint frame synchronization and frequency offset estimation algorithm suitable for tropospheric scatter communication, addressing the issues of multipath fading and frequency offset at both the transmission and reception ends. They employed a partial correlation frequency domain capture algorithm based on Fast Fourier Transform (FFT) to search for the maximum correlation value and frequency offset index, simultaneously achieving frame synchronization and frequency offset estimation [19]. The aforementioned algorithms rely on good communication conditions as well as sufficient received data which limits their application in the case of high bit error rates and limited intercepted data.

Convolutional Neural Networks (CNN) [20] are a type of feedforward neural network that includes convolutional computations and has a deep structure. The parameter sharing of convolutional kernels within the hidden layers and the sparsity of connections between layers enable CNNs to learn grid-like topological features, such as pixels and audio, with a smaller computational load. This leads to stable performance without requiring additional feature engineering, making them widely applicable in fields such as computer vision and natural language processing [21,22,23]. Some researchers have introduced deep learning and CNN into frame synchronization recognition [24,25,26,27]. Li utilized some one-dimensional data labeled with one-hot encoding to train the neural network and realized the synchronization recognition of equal-length frames [24]. Shao and Lei transformed digital sequences into images with column number equal to frame length and then trained the neural network to recognize the frame length [25]. Shen combined recurrent neural network (RNN) with the sliding window and periodic sampling, and implemented the recognitionof frame synchronization under Rayleigh fading channel conditions [26]. The data preprocessing process in [24,26] did not involve the idea of converting the sequence into an image, which did not adequately highlight the sample features. Moreover, the positive samples in [25] are limited to the case where the matrix column number is strictly equal to the frame length. Kojima et al. proposed a CNN-based symbol timing synchronization method for pilotless OFDM systems under severe multipath fading channels. By using a supervised CNN to classify the signals based on spectrograms, which contain power density information in both the time and frequency domains, simulation results show that this method can provide more accurate synchronization [27].

The Transformer architecture is a deep learning model designed for sequence data. It primarily utilizes a self-attention mechanism to capture long-range dependencies and allows for parallel processing of input, improving training efficiency. It excels at processing complete sequences of images by learning the global relationships within the sequence to update each patch. It has been widely applied in various fields such as image understanding, object detection, and semantic segmentation [28,29]. When frame-synchronized sequences are converted into matrices, the frame synchronization codes scattered across the frames are rearranged spatially, forming local stripe features. Depending on the number of columns in the matrix and the parameters of the frame synchronization sequence, the stripe width and inclination of the image samples generated from the matrix vary. Considering that CNNs are more adept at detecting simple features such as edges and lines, we have decided to conduct our research based on the CNN architecture.

Attracted by the stripes that appear when converting binary data with frame synchronization code into pictures and considering the powerful capability of CNN in image recognition, we propose a deep-learning-based blind frame synchronization identification method by combining the classical algorithms and deep learning in this paper. The binary data was firstly subjected to a flat-top interpolation operation [30]. Subsequently, it was transformed into a series of grayscale images and combined with scaling factors to generate RGB images. Then we use the image samples to train the convolutional neural network and employ the trained network to recognize the frame synchronization code in new binary sequences. Simulation results show that compared with the existing algorithms, the proposed algorithm has better recognition performance under the condition of higher bit error rate.

## 2. Algorithm Description

**Figure 1:**
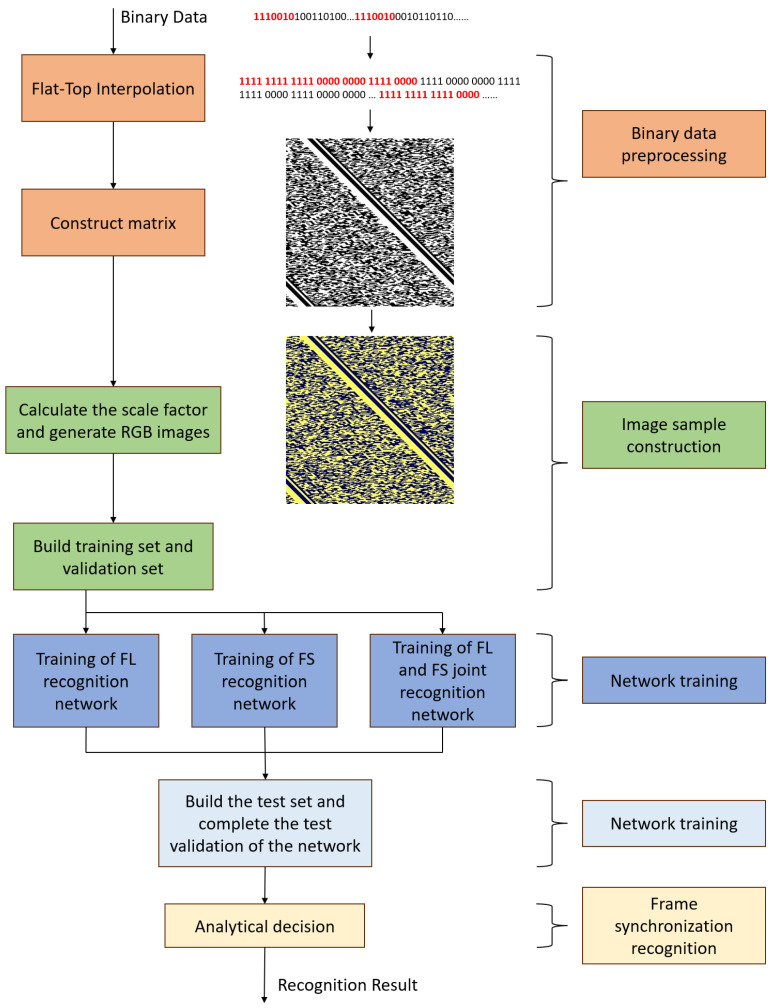
Frame synchronization code blind recognition algorithm flow based on deep learning. FL refers to Frame length and FS refers to Frame synchronization code.

The deep-learning-based blind frame synchronization recognition algorithm’s workflow is illustrated in Figure 1, which includes binary data preprocessing, image sample construction, frame synchronization recognition network training, network testing, and frame synchronization recognition. Firstly, in the preprocessing stage of binary data, we do a flat-top interpolation on the data and construct a series of matrixs with columns near the interpolated frame’s length and transform them into grayscale images. As the synchronization codes in different rows are only staggered by a small distance, the grayscale image shows obvious stripes. Then for each grayscale image, combine it with the scaling factor based on the size of the matrix to construct an RGB image, which is one of the samples in the data set. In the training stage, we label RGB images by their frame lengths, frame synchronization code types or both of them and train recognition networks for different labels. When a binary data sequence of unknown parameters is received, we use it to construct multiple RGB images of different sizes, and then send them to the trained network model for classification recognition, and obtain the classification results for the frame parameters of each image. Finally, we take the categories, quantities, and recognition probabilities into consideration to make a final recognition decision.

### 2.1. Binary Data Preprocessing

**Figure 2:**
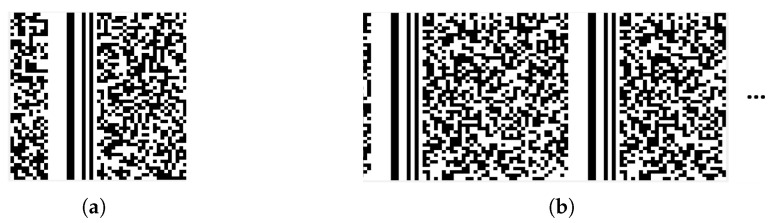
Vertical stripes when the number of matrix columns is equal to the frame length or an integer multiple of the frame length. (**a**) The number of columns equals the frame length. (**b**) The number of columns is an integer multiple of the frame length.

The frame length is defined as L=S+K, where *S* represents the length of the synchronization code and *K* represents the length of the data in one frame. Reshape a binary data sequence into a matrix *H* of size n×n, and when L|n, the synchronization code in each frame appear in the same column of the matrix. Then the column vectors of the matrix are divided into synchronization code columns and data bits columns. In this case, if the matrix is converted into a binary grayscale image, there will be distinct vertical stripes in the positions of the synchronization code columns, as shown in Figure 2.

**Figure 3:**
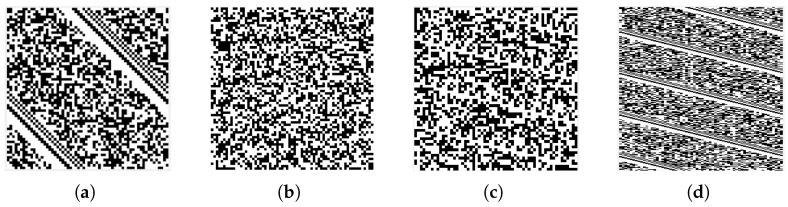
Images when the number of matrix columns is not equal to the frame length. (**a**) The number of columns is close to the frame length; (**b**) The number of columns differs greatly from frame length; (**c**) Image with faint stripes visible; (**d**) Images generated from a sequence using flat-topped interpolation.

When *n* is close to but not equal to *L*, the corresponding synchronization bits in each frame of the digital sequence are not in the same column. Figure 3a showns the case for n<L, each row of the matrix is not sufficient to accommodate a complete frame, the synchronization bits of the next frame will shift backward, eventually forming oblique stripes with a negative slope of 1/(n−L). It is similar for the the case of n>L, this time the value of the slope is positive. With the increase of the difference, the stripes will be extremely narrow, as shown in Figure 3c. To enhance the stripe features of the images, we perform a flat-top interpolation on the original binary data stream with an interpolation factor of 4. The reshaped image sample from interpolated data is shown in Figure 3d, the more pronounced stripes will facilate the processing later. Thus, the data preprocessing includes the flat-top interpolation and the reshape of the data after interpolation.

When *n* and *L* differ greatly, as shown in Figure 3b, the black and white pixels tend to be randomly distributed, there is no stripes in the image. The above two cases can be defined in the following mathematical way:

**Definition** **1.** *If the value of n is equal to or close to the length of the interpolated frame $L_{\alpha }$ with $L_{\alpha }=4L$, satisfying*

(1) $$0\leq |n-L_{\alpha }|\leq d.$$

*then we define that n **matches*** $L_{\alpha }$*, where d represents the **matching radius**. In this case, there are obvious striples in the sample. The case where Equation (1) is not satisfied is defined as n **dis-matches***$L_{\alpha }$*, there will be no striples in the sample.*

### 2.2. Image Sample Construction

According to Section *A*, when *n* matches $L_{\alpha }$, the constructed images will be used as positive samples; when *n* dis-matches $L_{\alpha }$, the obtained images will be used as negative samples. Negative samples also contain image samples constructed from randomly generated binary sequences to enhance the generalization ability of the network model.

The neural network we will use requires the input image size to be 224×224, so the grayscale images should be resized before being fed into the network. The interpolated frame length $L_{\alpha }$ can be obtained from the slope of the fringe $1/(n-L_{\alpha })$. However, the image scaling process will cause *n* lost. To solve this problem, except for the grayscale images we need to add additional parameters to compensate for the information loss during image scaling. We will use RGB images as the input of the neural network. Both R and G channels will be filled with the grayscale images. For B channel, we introduce a scaling factor to fill in each pixel. The scale factor is given by

(2) $$\beta =\frac{n}{224}.$$

where $\beta \in [\beta_{min},\beta_{max}]$ with

(3) $$\left\{\begin{array}{c} \beta_{min}=\frac{L_{min}-d}{224}.\\ \beta_{max}=\frac{L_{max}+d}{224}. \end{array}\right.$$

where $L_{min}$ and $L_{max}$ respectively represent the minimum $L_{\alpha }$ and the maximum $L_{\alpha }$ in the training set.

Considering that the data matrix is built from a binary sequence, it is necessary to map the value of β to [0, 1] using the following formula:

(4) $$\beta^{\prime }=\frac{\beta -\beta_{min}}{\beta_{max}-\beta_{min}}.$$

After the scaling process using bilinear interpolation, the original pixel values in the R and G channels will be stretched, leading to blurriness or distortion in the image, while the pixel values in the B channel remain the same and are not affected by the bilinear interpolation operation. As a result, frame length feature of the image, represented by the scale factor, are preserved.

**Figure 4:**
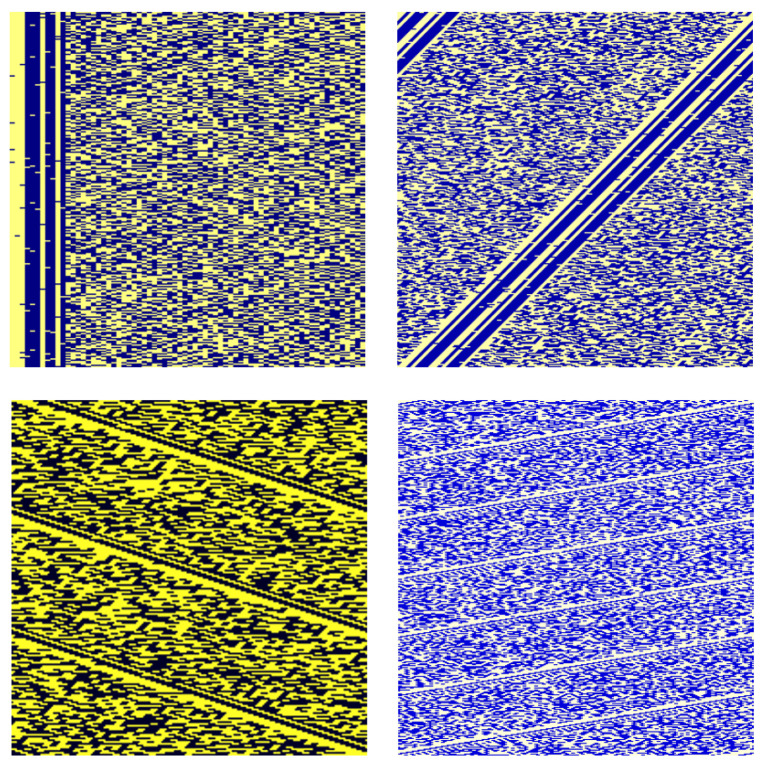
Some positive samples of the generated images.

Some positive samples are shown in Figure 4. These images show obvious stripes, and each row contains at most one complete frame, with scattered spots in the stripes due to the existence of error bits. In addition, RGB images with different scaling factors exhibit differences in color.

### 2.3. Frame Synchronization Recognition Network Training

Let’s denote the set of frame length categories as $\lambda =\{L_{1},L_{2},L_{3},\ldots ,L_{i},\ldots ,L_{p}\}$, where *p* represents the total number of frame length categories to be recognized. For each frame category to be recognized, we generated enough data frames according to the frame structure with error rate of 0.02m, m=[0,1,2,…,10], and then constructed positive and negative samples as described in Section *B*. All *n* satifies Equation (1) will be used to generated positive samples, so that all types of stripes will be included, the recognition ability of the network model will be improved. Each RGB image in positive samples is labeled with its corresponding frame length category. Split both the positive samples set and negative samples set with the same error rates into training and validation sets in a 9:1 ratio. All the training(validation) set for all the frame length categories with various error rates constitute the training(validation) set for the neural network.

CNN typically consists of multiple layers, where each layer sequentially performs operations such as convolution and pooling on the input information. This process ultimately completes the extraction of image features. The output of an image after passing through the convolutional layers can be written as

(5) $$Y_{i,j,k}=b\left(k\right)+\sum \sum \sum \left[X\left(m,n,c\right)W\left(k,m,n,c\right)\right].$$

Considering the wide application and diversified branches of ResNet in image classification, we choose to use ResNet50 network in our recognition algorithm. ResNet50 [31] introduces the structure of residual blocks, which successfully solves problems such as gradient vanishing and explosion in training deep neural networks. This enables the network to learn feature representations more deeply, thereby improving the model’s performance. Table 1 presents the network structure of the ResNet50 model used in this paper.

The activation function and loss function used in ResNet50 network are shown in Equations (6) and (7):

(6) ReLU(x)=max(0,x).

(7) $$C=-\frac{1}{n}\sum_{x}y_{i}lna_{i}.$$

where Equation (6) is ReLU function, and Equation (7) is cross-entropy loss function of multi-classification form.

We feed the training set into the neural network model and the model will iteratively update the network parameters with the stochastic gradient descent algorithm. The training process continues until the cross-entropy loss function of the network reaches its minimum value, resulting in a well-trained model for frame length recognition.

## 3. Test and Analysis

**Figure 5:**
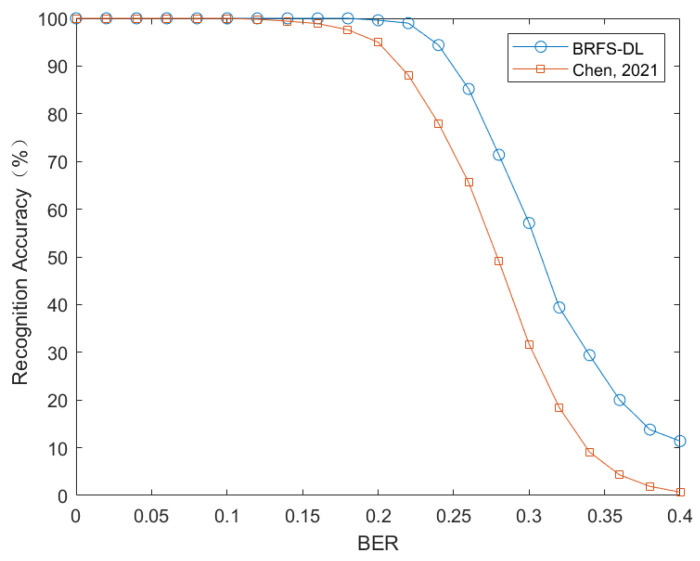
Comparison of frame length recognition performance between BRFS-DL and reference [12] (Chen, 2021).

To evaluate the ability of blind recognition algorithm for frame synchronization based on deep learning (BRFS-DL) to recognize frame lengths, the data set is configured with five different physical frame lengths of {50, 60, 70, 80, 90} and each frame length utilizes the same 11-bit Barker code as the synchronization code. When constructing the matrix *H*, the matching radius d=10, and the starting position of the extracted data sequence is randomly determined. The search range of $L_{\alpha }$ is set to [160, 400] considering the interpolation factor of 4. The simulation results are shown in Figure 5.

**Figure 6:**
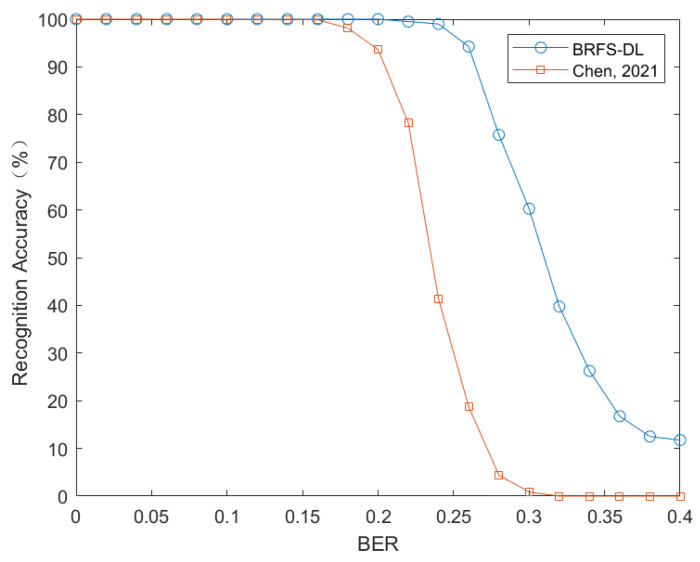
Comparison of frame synchronization code recognition performance between BRFS-DL and reference [12] (Chen, 2021).

When evaluating the ability of BRFS-DL to recognize frame synchronization codes, the data set is set up with four different synchronization codes with length of {7, 11, 13, 15}. Each category has the same frame length L=50, the number of columns of the matrix *H* is set to n∈[190,210]. The simulation results are shown in Figure 6.

**Figure 7:**
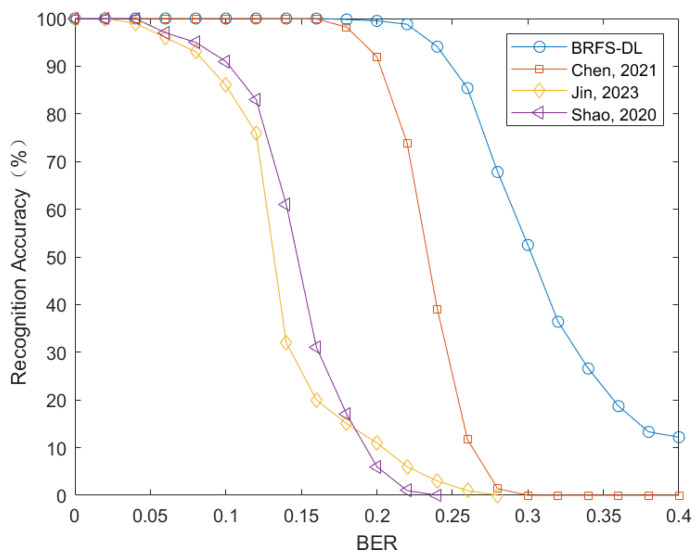
Comparison of joint recognition performance of frame length and frame synchronization code between BRFS-DL, references [12] (Chen, 2021), [13] (Jin, 2023) and [25] (Shao, 2020).

To verify whether BRFS-DL can simultaneously recognize the frame length and synchronization code, a data set is created with four different frame lengths {50, 60, 70, 80} and three different synchronization codes with length of {11, 13, 15}, totaling 12 different categories. A negative sample category is also included. The matrix *H* is constructed with d=10 and search range of $L_{\alpha }$ is set to [160,360]. The simulation results are shown in Figure 7.

Using three network models trained on the above three datasets for recognition prediction, the error rate of the original binary data sequence is set to 0.02m, m=[0,1,2,…,20], and each network was subjected to 200 random experiments for each error rate. In each experiment, the data sequence was first interpolated and then multiple matrices *H* were constructed using the same interpolated data sequence, with the number of columns of *H* increasing by $d_{i}=5$ within the range of *n*. Each matrix was transformed into an RGB image by combining its respective scaling factor, and fed into the trained network model for recognition. For every image, the recognition result includes a category and probability. We count the number of times ($N_{i}$) the sequence is recognized as being in each category ($C_{i}$) and the maximum probability ($P_{i}$) of being judged to be in that category, denoted by {$C_{1},N_{1},P_{1},C_{2},N_{2},P_{2},\ldots ,C_{p},N_{p},P_{p},$}. If there is only one category $C_{i}$ satisfying $N_{i}=max\left\{N_{1},N_{2},\ldots ,N_{p}\right\}$, then we take ($C_{i}$) as the recognition result. If there are multiple categories satisfying $N_{i}=max\left\{N_{1},N_{2},\ldots ,N_{p}\right\}$, the category with the highest classification probability among them is chosen as the recognition result for this experiment.

Following, we simulate our algorithm and compare it with the method proposed in reference [13,25] and reference [12] which is implementated with FPGA in reference [32]. Reference [12] constructed matrices with varying column numbers and computed their first-order cumulants. Decision thresholds were set based on frame structure characteristics to distinguish between synchronous bit columns and data bit columns, enabling recognition of frame length and synchronization codes. Reference [13] utilized high-order cumulant to detect the burst signal in time domain, and then completed the frame synchronization recognition by combining the code domain information. Reference [25] transformed digital sequences into images with column number equal to frame length and then trained the neural network to recognize the frame length.

The frame length recognition results are shown in Figure 5. When the error rate is less than 0.2, BRFS-DL achieves a recognition accuracy of 100%. Furthermore, even when the error rate is as high as 0.25, the recognition accuracy of BRFS-DL remains above 90%.

Figure 6 shows the performance comparison between BRFS-DL and the algorithm in reference [12] in frame synchronization code recognition. It can be observed that when the error rate is less than 0.2, the recognition accuracy of BRFS-DL algorithm reaches 100%. In addition, even when the error rate is up to 0.25, the recognition accuracy of BRFS-DL algorithm is still above 95%.

The performance comparison of the four methods for comprehensive recognition of frame length and frame synchronization code is depicted in Figure 7. When the error rate is 0.15, the blind recognition method of frame synchronization in time-code domain proposed in Reference [13] achieves a recognition probability of about 22%, and the convolutional neural network-based physical layer cutting method in Reference [25] has a recognition probability of about 40%. In contrast, our algorithm achieves a recognition accuracy of 100%. Moreover, when the error rate is around 0.25, the recognition accuracy still exceeds 90%. In contrast, the first-order cumulant and error elimination method presented in reference [12] exhibits a rapid decline in recognition performance at this error rate and fails to accurately recognize frame synchronization. The main reason for the good recognition performance of BRFS-DL is that it transforms the feature of frame length into a scaling factor and combines it with the sequence to create a striped RGB image for neural network training. By employing flat-top interpolation on the original data and setting an appropriate matching radius *d*, the diversity of the dataset is enhanced. In contrast, the recognition methods in references [12,13] require setting suitable thresholds to make decisions based on the computed first or higher-order cumulants, however, it is difficult to find a threshold that fits all scenarios at the same time. Furthermore, the recognition method in reference [13] relies on the autocorrelation characteristics of frame synchronization codes to correct the data in the analysis matrix, which limits its applicability in high bit error rate environments. Reference [25] also employs the idea of converting sequences into images and using neural networks for recognition. However, it requires that the number of columns in the matrix be strictly uniform with the frame length during data compression, which is equivalent to setting the matching radius to 0 in our method. Additionally, it lacks the process of flat-top interpolation. Therefore, our method improves recognition accuracy while reducing computational complexity.

In constructing the training set, we used a matching radius of *d* and column number increment of 1, which means that all the matrices whose column number is within the range [L−d,L+d] are used to generate positive samples. In each experiment, we generate multiple images of varying sizes for the fixed binary sequence. Theoretically, as long as one of the images has a matrix column number within the range [L−d,L+d], we can obtain the correct recognition result. In realization, the increment of the matrix column number $d_{i}$ is less than *d*, which means that at least two positive samples will definitely appear in one experiment, providing us with more grounds for post-selection, enabling us to reach more accurate decision results. It is obvious that the recognition accuracy will increase as $d_{i}$ decreases, but this comes at the cost of increased computational complexity. It’s a trade-off between computational complexity and accuracy.

Even with the maximum $d_{i}=2d+1$, the computational complexity of our method will still be significantly higher than the traditional algorithms, due to the millions of parameters in the neural network model. For example, ResNet-50 has approximately 25.6 million parameters and a computational complexity of about 4.1 billion floating point operations (FLOPs), whereas the complexity of the algorithm in reference [12] is approximately O(n), where *n* is the actual frame length. Given that blind recognition is always used in a non-cooperative communication scenario with higher BER and limited prior information, it is more reasonable to sacrifice computational complexity to achieve better recognition performance. The main purpose of this paper is to demonstrate that using neural networks for frame synchronization recognition can achieve good performance. Further we can focus on the complexity and recognition performance of the algorithm to obtain neural networks with low complexity and excellent performance.

## 4. Conclusions

In order to improve the error tolerance of classical blind frame synchronization recognition algorithm, we proposed a BRFS-DL algorithm based on deep learning. Inspired by the property that RGB images have distinct stripes when the number of columns of the matrix matches the frame length, we generate positive and negative samples to train the neural network model. Moreover, we interpolated the original Binary data in order to enhance the stripe features and combined the scaling factor to improve the recognition accuracy. Three recognition networks, the frame length recognition network, frame synchronization code recognition network, and combined frame length and frame synchronization code recognition network are separately trained. Finally, the recognition networks are tested and verified using a test set. Simulation results demonstrate that compared with the algorithm proposed in reference [12], BRFS-DL algorithm achieves higher recognition accuracy. Even at an error rate of 0.25, the recognition accuracy still exceeds 90%, BRFS-DL algorithm exhibits significant improvements in error tolerance.

Nevertheless, our current algorithm is still unable to operate in a completely non-cooperative communication environment without any prior information. The trained neural network model can only recognize a limited number of predefined frame synchronization categories, indicating insufficient generalization capability. Moreover, in order to simplify the data preprocessing steps, we intentionally discarded several operations that could highlight the features of the frame synchronization code, such as gradient computation and Fourier transform. This may, to some extent, limit the further improvement of the algorithm’s performance. In addition, our algorithm has only experimented with CNN networks. Future work will consider architectures such as recurrent neural network(RNN) and Transformers, with a greater focus on enhancing the model’s generalization ability and optimizing data preprocessing steps, so that our algorithm can be more effectively applied in practical non-cooperative communication scenarios.

## Figures and Tables

**Table 1:** ResNet50 Network Structure.

Layer Name	Output Size	Parameters for Each Layer
Conv1	112 × 112	7 × 7, 64, stride2
Conv2_x	56 × 56	3 × 3 Max_Pooling, stride2 $\left[\begin{array}{c} 1\times 1,64\\ 3\times 3,64\\ 1\times 1,256 \end{array}\right]\times 3$
Conv3_x	28 × 28	$\left[\begin{array}{c} 1\times 1,128\\ 3\times 3,128\\ 1\times 1,512 \end{array}\right]\times 4$
Conv4_x	14 × 14	$\left[\begin{array}{c} 1\times 1,256\\ 3\times 3,256\\ 1\times 1,1024 \end{array}\right]\times 6$
Conv5_x	7 × 7	$\left[\begin{array}{c} 1\times 1,512\\ 3\times 3,512\\ 1\times 1,2048 \end{array}\right]\times 3$
	1 × 1	Average pool, 1000d fc, Softmax

## Data Availability

Data are contained within the article.

## References

[1] Chiani M., Martini M.G. (2005). Practical frame synchronization for data with unknown distribution on AWGN channels. IEEE Commun. Lett..

[2] Gan H.Q., Wang J., Zhou P., Li S.Q. (2015). An Antijaming OFDM Synchronization Algorithm under Complicated Multipath Fading Channels. Signal Process..

[3] Zhang Y., Yang X.J. (2013). Recognition Method of Concentratively Inserted Frame Synchronization. Acta Armamentarii.

[4] Liang Y., Rajan D., Eliezer O.E. (2015). Sequential Frame Synchronization Based on Hypothesis Testing with Unknown Channel State Information. IEEE Trans. Commun..

[5] He X.D., Tan X.D., Wu B.Y. (2022). Wireless Clock Synchronization System Based on Long-wave Signal Sensing. Proceedings of the 2022 41st Chinese Control Conference (CCC).

[6] Wang D., Xie H., Wang F.H., Huang Z.T. (2013). Blind identification of frame synchronization codes based on first-order cumulant. Commun. Countermeas..

[7] Qin J., Huang Z., Liu C., Su S., Zhou J. (2015). Novel blind recognition algorithm of frame synchronization words based on soft-decision in digital communication systems. PLoS ONE.

[8] He J., Zhou J.R. (2017). Improvement of Frame Synchronization Algorithm for Burst Data in Anti-interference Network. Radio Commun. Technol..

[9] Xu Y.Y., Zhong Y., Huang Z.P. (2019). An improved blind recognition algorithm of frame parameters based on self-correlation. Information.

[10] Shao K., Lei Y.K. (2020). Fast Blind Recognition Algorithm of Frame Synchronization Based on Dispersion Analysis. J. Signal Process..

[11] Kil Y.S., Lee H., Kim S.H., Chang S.H. (2020). Analysis of blind frame recognition and synchronization based on sync word periodicity. IEEE Access.

[12] Chen X.F., Liu N.N., Xu W.B. Low Complexity Blind Recognition Method for Frame Synchronization. Proceedings of the 14th National Conference on Signal and Intelligent Information Processing and Application.

[13] Jin M., Zhang S., He X., Quan D., Wei J. (2023). Blind Recognition of Frame Synchronization in Time-Code Domain. Proceedings of the 2023 International Conference on Ubiquitous Communication (Ucom).

[14] Imad R., Houcke S. (2021). On blind frame synchronization of LDPC codes. IEEE Commun. Lett..

[15] Xia T., Wu H.C., Chang S.Y. (2014). Joint blind frame synchronization and encoder identification for LDPC codes. IEEE Commun. Lett..

[16] Feng Z., Liu Y., Zhang S., Xiao L., Jiang T. (2023). Polar-Coding-Assisted Blind Frame Synchronization Based on Soft Information of Frozen Bits. IEEE Commun. Lett..

[17] Wang Z., Zhang S., Sun H., Gong K., Liu H., Wang W. Fast Detection Algorithm for Equal Length Frame Signals Based on Pattern Matching. Proceedings of the 2nd International Conference on Signal Processing, Computer Networks and Communications.

[18] Wang Y.Q., Hu P.J., Yang J.A. (2024). Blind synchronization word recognition algorithm for non-equal length frame based on two window-sliding operations. Syst. Eng. Electron..

[19] Lv Z.H., Zhang T., Ren W.C. (2024). Study on Joint Frame Synchronization and Frequency Bias Estimation Algorithm for Tropospheric Scattering Channels. Comput. Meas. Control..

[20] LeCun Y., Boser B., Denker J., Henderson D., Howard R., Hubbard W., Jackel L. (1989). Handwritten digit recognition with a back-propagation network. Adv. Neural Inf. Process. Syst..

[21] Chen C., Wang W., Liu Z., Wang Z., Li C., Lu H., Pei Q., Wan S. (2024). RLFN-VRA: Reinforcement Learning-based Flexible Numerology V2V Resource Allocation for 5G NR V2X Networks. IEEE Trans. Intell. Veh..

[22] Chen C., Si J., Li H., Han W., Kumar N., Berretti S., Wan S. (2024). A High Stability Clustering Scheme for the Internet of Vehicles. IEEE Trans. Netw. Serv. Manag..

[23] Xiao T., Chen C., Pei Q., Jiang Z., Xu S. (2023). SFO: An adaptive task scheduling based on incentive fleet formation and metrizable resource orchestration for autonomous vehicle platooning. IEEE Trans. Mob. Comput..

[24] Li Y.H. (2020). Research on Non-Cooperative Signal Link Layer Analysis Technology. Master’s Thesis.

[25] Shao K., Lei Y.K. (2020). Physical Frame Segmentation Method Based on Convolutional Neural Network. J. Data Acquis. Process..

[26] Shen B.X. (2022). Noncooperative Signal Analysis via Deep Learning. Master’s Thesis.

[27] Kojima S., Goto Y., Maruta K., Sugiura S., Ahn C.J. (2023). Timing synchronization based on supervised learning of spectrogram for ofdm systems. IEEE Trans. Cogn. Commun. Netw..

[28] Ning E., Zhang C., Wang C., Ning X., Chen H., Bai X. (2023). Pedestrian Re-ID based on feature consistency and contrast enhancement. Displays.

[29] Wang C., Wu M., Lam S.K., Ning X., Yu S., Wang R., Li W., Srikanthan T. (2024). GPSFormer: A Global Perception and Local Structure Fitting-based Transformer for Point Cloud Understanding. arXiv.

[30] Alrubei M.A., Dmitrievich P.A. (2023). An Approach for Single-Tone Frequency Estimation Using DFT Interpolation with Parzen Windowing. Kufa J. Eng..

[31] He K., Zhang X., Ren S., Sun J. Deep residual learning for image recognition. Proceedings of the IEEE Conference on Computer Vision and Pattern Recognition.

[32] Liu N.N. (2021). Research and FPGA Implementation of Blind Synchronization Algorithm. Master’s Thesis.

